# Machine Learning and Molecular Modeling Strategy for the Identification of CNS-Active Acetylcholinesterase Inhibitors

**DOI:** 10.3390/ph19071120

**Published:** 2026-07-20

**Authors:** Muhammad Yasir, Jinyoung Park, Eun-Taek Han, Won Sun Park, Jin-Hee Han, Jongseon Choe, Wanjoo Chun

**Affiliations:** 1 Department of Pharmacology, Kangwon National University School of Medicine, Chuncheon 24341, Republic of Korea; yasir.khokhar1999@gmail.com (M.Y.); jinyoung0326@kangwon.ac.kr (J.P.); 2 Department of Medical Environmental Biology and Tropical Medicine, Kangwon National University School of Medicine, Chuncheon 24341, Republic of Korea; ethan@kangwon.ac.kr (E.-T.H.); han.han@kangwon.ac.kr (J.-H.H.); 3 Department of Physiology, Kangwon National University School of Medicine, Chuncheon 24341, Republic of Korea; parkws@kangwon.ac.kr; 4 Department of Microbiology and Immunology, Kangwon National University School of Medicine, Chuncheon 24341, Republic of Korea; jchoe@kangwon.ac.kr

**Keywords:** acetylcholinesterase inhibitors, enamine compounds, BBB permeable compounds for AD, molecular docking, molecular dynamic simulation

## Abstract

**Background:** Acetylcholinesterase (AChE) is a key therapeutic target in neurological disorders, and the discovery of novel inhibitors with improved efficacy and pharmacokinetic properties remains a significant challenge. **Methods:** In this study, an integrated computational and experimental approach was employed to identify potential AChE inhibitors. A machine learning-based model was developed to predict bioactive compounds from large chemical libraries, followed by Blood–Brain Barrier (BBB) permeability screening to ensure Central Nervous System (CNS) suitability. The shortlisted compounds were further evaluated using molecular docking, molecular dynamics simulations, and MM-PBSA binding free-energy calculations to assess their interaction profiles and stability. Selected top-ranked compounds were subjected to in vitro biological evaluation for AChE inhibitory activity. **Results:** The results demonstrated that several screened compounds, including Z1498348710 and Z1824281875, exhibited notable inhibition with AChE activity reduced to approximately 75% and 72%, respectively, compared to the control. Other compounds such as Z29542160, Z105150208, Z94570687, and Z94570675 showed moderate inhibitory effects, maintaining AChE activity in the range of 82–86%. In comparison, the reference inhibitors Donepezil and Neostigmine bromide displayed significantly stronger inhibition, reducing AChE activity to approximately 20% and 18%, respectively. **Conclusions:** Overall, the identified compounds demonstrated moderate AChE inhibitory activity while exhibiting favorable predicted physicochemical and computational profiles. These findings suggest that they represent promising starting points for medicinal chemistry optimization and future development as CNS-active AChE inhibitors.

## 1. Introduction

Alzheimer’s disease (AD) is a progressive neurodegenerative disorder characterized by a decline in cognitive function, memory, and behavioral abilities, ultimately leading to severe impairment in daily activities [1,2,3]. It is one of the most prevalent forms of dementia worldwide and represents a major public health challenge [4]. The pathogenesis of AD is complex and multifactorial, involving the accumulation of amyloid-β plaques, formation of neurofibrillary tangles, oxidative stress, and neurotransmitter imbalance [5,6,7]. Among these, a marked reduction in cholinergic neurotransmission, particularly decreased levels of acetylcholine (ACh), is strongly associated with cognitive decline in AD patients [8].

Acetylcholinesterase (AChE; EC 3.1.1.7) is a key enzyme responsible for the rapid hydrolysis of Acetylcholine (Ach) in synaptic clefts, thereby regulating cholinergic signaling [9,10,11]. Inhibition of AChE has been widely recognized as an effective therapeutic strategy for enhancing cholinergic transmission and improving cognitive symptoms in AD [12,13,14]. Consequently, several AChE inhibitors have been developed and approved for clinical use, including Donepezil, which remains one of the most commonly prescribed drugs for symptomatic management [15,16]. Despite its clinical utility, Donepezil is associated with several limitations. It primarily offers symptomatic relief without halting or reversing disease progression [17,18]. In addition, its use is often accompanied by adverse effects such as gastrointestinal disturbances, insomnia, dizziness, and cardiovascular complications, including bradycardia. Variability in patient response and limited selectivity further constrain its therapeutic efficacy [19,20,21]. These limitations underscore the need for the development of novel AChE inhibitors with improved potency, selectivity, safety, and pharmacokinetic profiles.

A major challenge in the development of central CNS-active therapeutics is the ability of compounds to effectively cross the Blood–Brain Barrier (BBB), which restricts the entry of many pharmacologically active molecules into the brain [22,23,24]. Therefore, identifying compounds that combine strong target affinity with favorable BBB permeability is essential for successful drug discovery.

Recent advances in artificial intelligence (AI) and computational drug discovery have significantly accelerated early-stage hit identification by enabling the rapid screening of millions of compounds while reducing the time and cost associated with conventional high-throughput screening [25,26,27,28,29,30]. Although numerous computational studies have reported virtual screening of acetylcholinesterase inhibitors, many rely primarily on molecular docking as the initial screening strategy, which is computationally demanding for ultra-large chemical libraries and may generate high false-positive rates when used alone.

In this study, we developed a multi-stage computational screening pipeline designed to improve both screening efficiency and hit quality for the discovery of novel AChE inhibitors. A machine learning (ML) model trained on experimentally reported AChE inhibitors was first employed to prioritize active compounds from a large chemical library, substantially reducing the number of candidates requiring computationally intensive structure-based analyses. To improve the translational relevance of the identified hits predicted active compounds were subsequently filtered based on blood–brain barrier permeability to enrich molecules with favorable CNS exposure. The remaining candidates were then evaluated using molecular docking, molecular dynamics simulations, and MM-PBSA binding free-energy calculations to investigate binding affinity and complex stability. Finally, the highest-ranked compounds were experimentally evaluated to validate their inhibitory activity against AChE. This integrated strategy differs from many previously reported AChE virtual screening studies by combining machine learning-guided prioritization, pharmacokinetic filtering, comprehensive structural validation, and biological verification within a single discovery framework.

## 2. Results

### 2.1. Dataset Characteristics and Chemical Space

**Figure 1:**
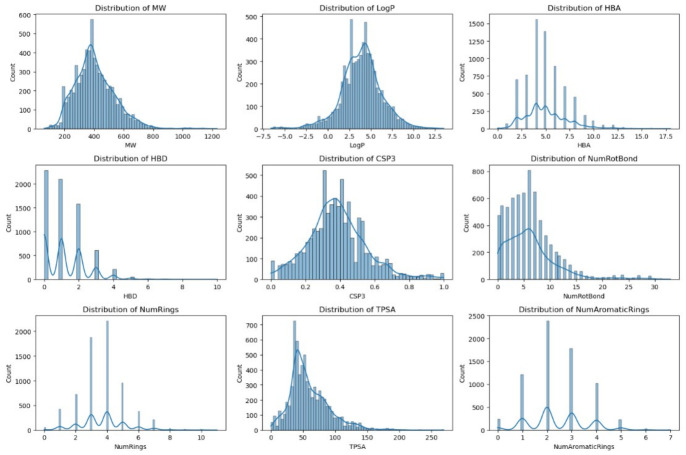
Distribution profiles of key physicochemical properties of the screened compound library, including molecular weight (MW), lipophilicity (LogP), hydrogen bond acceptors (HBA), hydrogen bond donors (HBD), fraction of sp^3^ carbons (CSP3), number of rotatable bonds (NumRotBond), total ring count (NumRings), topological polar surface area (TPSA), and number of aromatic rings (NumAromaticRings), highlighting the chemical diversity and drug-likeness of the dataset.

The curated AChE inhibitor dataset exhibited a broad range of bioactivity values, highlighting the diverse potency profiles of the compounds included in the study. Analysis of the physicochemical descriptor distributions revealed substantial variability in key molecular properties, particularly molecular weight and lipophilicity (LogP), indicating that the dataset encompasses compounds spanning a wide chemical space. The distribution of topological polar surface area (TPSA) suggested a moderate range of polarity, while the values for hydrogen bond donors and acceptors reflected a relatively balanced hydrogen bonding capacity across the dataset (Figure 1). Such diversity in physicochemical characteristics is advantageous for developing predictive models with improved generalizability.

Further insights were obtained from correlation analysis, which demonstrated a moderate positive relationship between molecular weight and lipophilicity, consistent with general chemical trends where larger molecules tend to exhibit increased hydrophobicity. In contrast, most other descriptors showed weak intercorrelations, suggesting minimal redundancy among the selected features (Supplementary Data Figure S1). This low level of multicollinearity indicates that each descriptor contributes unique and complementary information to the model. Collectively, the observed diversity and independence of the descriptors enhance the robustness of the dataset and support the development of reliable machine learning models for predicting AChE inhibitory activity.

### 2.2. Model Performance and Feature Importance Insights

The Random Forest regression model demonstrated good predictive performance in estimating the inhibitory activity of the compounds against AChE. A high degree of correlation was observed between the predicted and experimentally determined pIC_50_ values, indicating that the model was able to effectively capture the underlying structure–activity relationships within the dataset. In addition, the model performance of the test set exhibited:

- Mean Squared Error (MSE) = 0.84
- Mean Absolute Error (MAE) = 0.54
- R2 = 0.65

**Figure 2:**
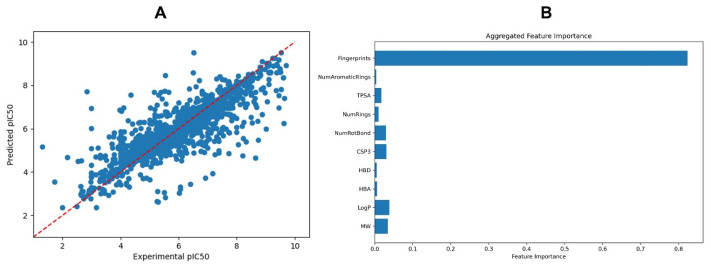
(**A**) Correlation between experimentally determined and model-predicted pIC_50_ values, demonstrating the predictive performance of the machine learning model (red dashed line indicates ideal fit). (**B**) Aggregated feature importance analysis highlighting the relative contribution of molecular descriptors and fingerprints to the model’s predictions.

Reflecting minimal prediction deviations and overall reliability. Evaluation on the independent test set further confirmed the model’s robustness, as it maintained good generalization performance on previously unseen data, suggesting that overfitting was adequately controlled. Visual assessment using scatter plots of predicted versus experimental pIC_50_ values provided additional support for the model’s accuracy. The majority of data points were distributed closely along the ideal diagonal line (y = x), demonstrating strong agreement between predicted and actual values (Figure 2).

Feature importance analysis of the Random Forest model provided valuable insights into the key molecular determinants governing AChE inhibitory activity. Among the calculated descriptors, topological polar surface area (TPSA) emerged as the most influential feature, followed by lipophilicity (LogP), fraction of sp^3^-hybridized carbons (CSP3), and molecular weight. The prominence of TPSA and LogP highlights the critical role of molecular polarity and lipophilicity in modulating ligand–target interactions, particularly in the context of AChE, where an optimal balance between hydrophilicity and hydrophobicity is essential for effective binding. Additionally, the contribution of CSP3 and molecular weight suggests that structural complexity, flexibility, and degree of saturation also play important roles in determining biological activity.

Beyond physicochemical descriptors, molecular fingerprints were found to collectively contribute significantly to the model’s predictive capability. Interestingly, the top 10 bits contribute: 0.3481 (42.3% of total FP importance) (Table 1). These fingerprints encode detailed structural information, enabling the model to capture subtle variations in chemical architecture that are not fully described by global descriptors alone. Individual fingerprint bits exhibited varying levels of importance, reflecting their ability to represent specific substructures or functional groups associated with AChE inhibition. Together, the combined influence of descriptors and fingerprints underscores the importance of integrating both physicochemical properties and structural features to achieve accurate and interpretable predictive models.

### 2.3. Virtual Screening Outcomes

Application of the trained machine learning model to a large enamine screening library enabled the efficient identification of potent AChE inhibitors. The distribution of predicted pIC_50_ values across the screened compounds exhibited an approximately normal pattern, indicating a balanced spread of predicted activities within the chemical space. Notably, one compound demonstrated predicted potency with pIC_50_ values greater than 8, while several compounds manifested high pIC_50_ values above 7, worthy of further consideration (Supplementary Data Figure S2).

To ensure the reliability and uniqueness of the selected candidates, duplicate entries were systematically removed based on their SMILES representations. This refinement step resulted in a curated list of unique, high-confidence hits with strong predicted inhibitory potential. The top-ranked compounds were subsequently subjected to structural visualization, which revealed a diverse range of chemical scaffolds and functional groups (Supplementary Data Figure S3). This observed chemical diversity is particularly important, as it increases the likelihood of identifying novel scaffolds with favorable pharmacological properties and provides a robust foundation for further computational analysis.

### 2.4. Structural Analysis of the AChE Protein

**Figure 3:**
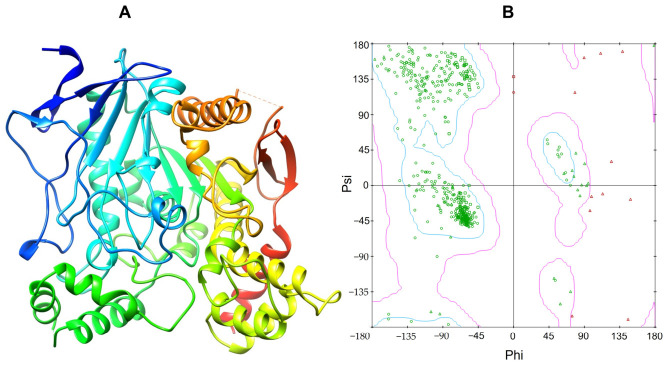
(**A**) Three-dimensional structure of acetylcholinesterase (AChE) displayed as a rainbow-colored cartoon, with the N-terminus shown in blue and the C-terminus in red, illustrating the overall protein fold and secondary structural elements. (**B**) Ramachandran plot showing the distribution of backbone dihedral angles (Psi and Phi), where green circles represent residues in favored regions, red triangles indicate residues in disallowed regions, and the blue and magenta contour lines denote the favored and allowed conformational regions, respectively.

The X-ray crystallographic structure of AChE (PDB ID: 7E3H) with a resolution of 2.45 Å was retrieved from the Protein Data Bank (PDB). This high-resolution structure provides detailed information regarding the spatial arrangement of amino acid residues within the active site and the overall protein architecture. The catalytic machinery of the enzyme consists of a conserved catalytic triad, which is directly involved in the hydrolysis of acetylcholine. The VADAR v1.8 analysis revealed that the X-ray structure of AChE is composed of 34% helix, 23% β sheets, 41% coils, and 23% turns. Furthermore, the Ramachandran analysis revealed that 97.2% of all residues were in the favored region, while 100% of all residues were in the allowed region (Figure 3), and there were no outliers.

### 2.5. The Binding Pocket Analysis

**Figure 4:**
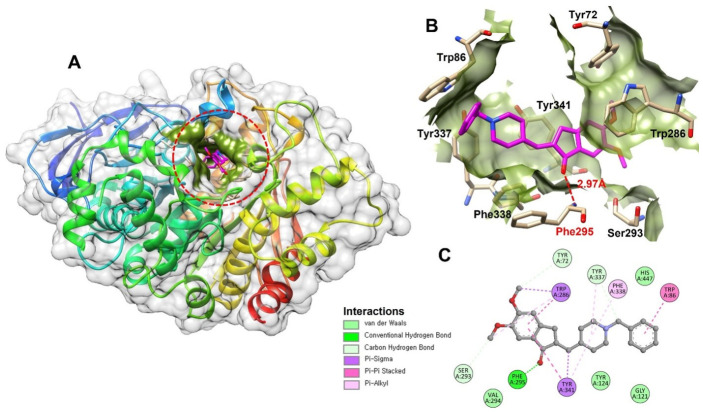
(**A**) Binding mode of the co-crystallized ligand within the active site of AChE, highlighting the binding pocket (red dashed circle) (white molecular surface, rainbow-colored protein cartoon, green binding pocket, and magenta ligand). (**B**) Close-up view of key ligand–residue interactions, showing hydrogen bonding with critical amino acids. The co-crystallized ligand was colored pink, while the interacting amino acids are colored tan. The hydrogen bonds and hydrogen bond forming amino acids are labeled in red. (**C**) Two-dimensional interaction diagram illustrating the network of interactions stabilizing the ligand within the active site.

The catalytically active site of AChE is located at the base of a deep and narrow active-site gorge, which is lined predominantly with aromatic amino acid residues that facilitate substrate recognition and stabilization. This active site is highly conserved and is responsible for the rapid hydrolysis of the neurotransmitter acetylcholine into choline and acetate, thereby terminating synaptic transmission. The active binding pocket of AChE was examined to characterize its physicochemical properties and key amino acid residues responsible for ligand interaction. The co-crystallized ligand (PDB ID: 7E3H) was utilized for the binding pocket assessment. Therefore, the number of key interacting amino acid residues was assessed, including Trp286, Tyr341, Trp86, Phe338, Phe295, Phe293, Tyr72, and Tyr337 (Figure 4). Among these, the amino acid Phe295 formed a hydrogen bond, and the other amino acids were involved in other van der Waals and hydrophobic interactions.

### 2.6. Molecular Docking Analysis

The Discovery Studio’s CDocker module facilitated the prediction of negative energy values, which included both the CDocker energy and the CDocker interaction energy. The CDocker energy is an indicator of the total docking energy, taking into account the 3D structure and physicochemical characteristics of both the ligand and the protein. On the other hand, the CDocker interaction energy focuses on the energy related to the interactions between the ligand and the receptor, including various intermolecular forces such as hydrogen bonds, electrostatic interactions, and van der Waals forces. The molecular docking results obtained using the Discovery Studio CDocker module revealed significant variation in binding energies among the screened compounds, indicating differences in their binding affinity and interaction stability within the active site of AChE.

Among the screened compounds, Z94570687 demonstrated the most favorable CDocker energy (−43.6447 kcal/mol), closely followed by Z94570693 (−43.3405 kcal/mol), Z94570675 (−43.0556 kcal/mol), and Z1824281875 (−42.9042 kcal/mol). The highly negative CDocker energy values of the top-ranked compounds indicate a strong binding trend across this subset, which may be attributed to favorable interactions such as hydrogen bonding, hydrophobic contacts, and π–π stacking within the active site.

Compounds such as Z260430114 (−40.5476 kcal/mol), Z29542160 (−40.0182 kcal/mol), and Z94570691 (−39.7155 kcal/mol) also exhibited robust binding affinities, remaining significantly superior to the reference compound. Even compounds with comparatively higher (less negative) energies within the screened set, such as Z238483938 (−33.9650 kcal/mol), Z86312003 (−33.7623 kcal/mol), and Z2047906229 (−33.1509 kcal/mol), still outperformed Donepezil by a considerable margin (Table 2).

Interestingly, while Donepezil displayed a relatively moderate overall CDocker energy, it manifested strong CDocker interaction energy (−56.0576 kcal/mol) compared to the top-ranked screened compounds, suggesting that despite its conformational positioning may reduce overall binding efficiency it still can make good interactions with the active site residues of the target protein. In contrast, several screened compounds achieved a better balance between interaction energy and conformational stability, resulting in significantly improved overall docking scores. For example, Z1824281875 and Z1498348710 exhibited highly favorable interaction energies (−56.8551 and −58.5919 kcal/mol, respectively), coupled with strong overall CDocker energies, reinforcing their potential as high-affinity binders.

### 2.7. BBB Permeability Evaluation

The integration of molecular docking results with BBB permeability predictions enabled a more comprehensive assessment of compound suitability for central nervous system targeting. By combining binding affinity with pharmacokinetic properties, the merged dataset allowed more informed prioritization of candidate molecules. Docking analysis revealed a range of binding strengths, with highly negative scores indicating strong ligand–protein interactions; however, these scores alone do not account for a compound’s ability to penetrate the BBB. Incorporation of BBB predictions addressed this limitation by introducing an additional selection criterion based on CNS permeability. These results showed that most of the top-ranked compounds in molecular docking manifested higher predicted BBB permeability (Table 3), suggesting their potential as viable candidates following further optimization. The application of combined filtering criteria further reinforces that the screened compounds may have the potential to inhibit the target protein in the CNS.

### 2.8. Molecular Interaction Analysis

**Figure 5:**
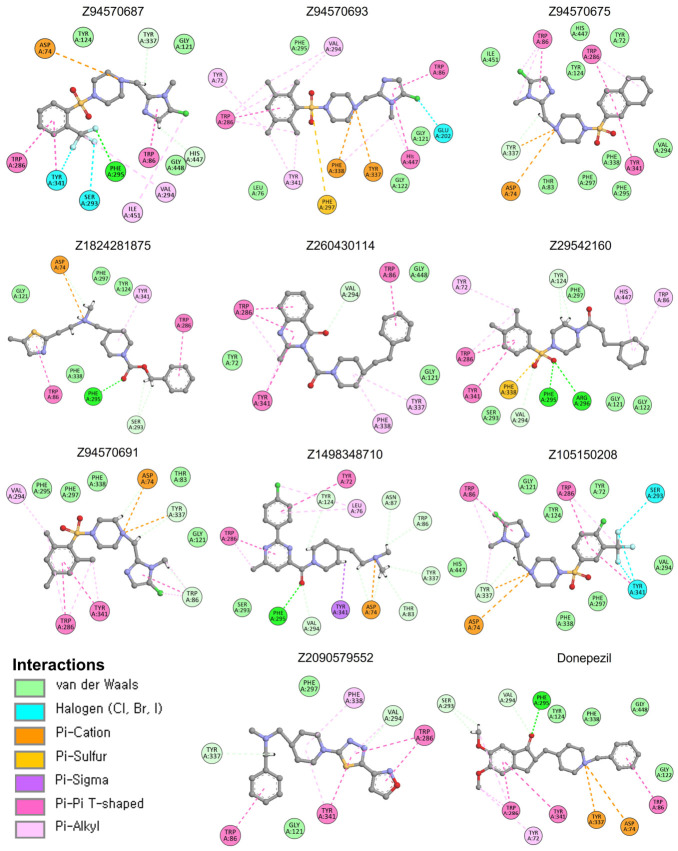
Two-dimensional interaction analysis of the top-ranked protein–ligand complexes, illustrating key non-covalent interactions such as hydrogen bonds, hydrophobic contacts, π–π stacking, and electrostatic interactions contributing to ligand binding within the active site.

The molecular interaction analysis of the top-ranked compounds within the AChE binding pocket (Figure 5) revealed a consistent interaction pattern dominated by hydrophobic contacts, including π–π stacking, π–alkyl, and van der Waals interactions with key aromatic residues such as Trp86, Trp286, Tyr337, Tyr341, Phe295, and Phe338. These residues, which line the active-site gorge, play a crucial role in stabilizing ligand binding through non-polar interactions. Notably, most compounds exhibited minimal hydrogen bonding, with only occasional contributions from residues such as Ser293, Phe295, or Tyr.

This interaction profile strongly correlates with the predicted BBB permeability scores obtained in this study. Compounds demonstrating higher predicted BBB scores predominantly favored hydrophobic interactions over polar contacts, reflecting lower topological polar surface area (TPSA) and reduced hydrogen bonding capacity. Since excessive hydrogen bonding and polarity are known to hinder passive diffusion across the BBB, the observed interaction patterns suggest that these ligands possess physicochemical properties favorable for CNS penetration. The limited number of hydrogen bonds, combined with dominant hydrophobic interactions, indicates that the compounds maintain an optimal balance between binding affinity and lipophilicity. This balance is critical, as it allows efficient accommodation within the hydrophobic AChE gorge while simultaneously supporting membrane permeability. In contrast to highly polar ligands, which may exhibit strong binding but poor BBB penetration, the selected compounds demonstrate a more drug-like profile suitable for CNS targeting.

Furthermore, the interaction similarity between the screened compounds and the reference drug Donepezil reinforces their potential as effective AChE inhibitors. Donepezil is known to rely heavily on aromatic and hydrophobic interactions within the binding gorge [31], and a comparable interaction pattern was observed for the majority of the identified hits. Overall, the integration of BBB scoring with molecular interaction analysis highlights that the selected compounds not only exhibit favorable binding within the AChE active site but also possess physicochemical characteristics conducive to BBB permeability. This dual advantage suggests that these candidates may effectively reach the central nervous system and inhibit the target protein without significant permeability limitations, making them promising leads for further analysis.

### 2.9. Molecular Dynamics Simulation

#### 2.9.1. RMSD

The root mean square deviation (RMSD) analysis was performed to evaluate the structural stability of the protein–ligand complexes over the simulation period, with lower RMSD values and smaller fluctuations indicating greater stability. The AChE 100 ns MD simulation (without ligand) was also carried out to observe the backbone stability of the target protein (Supplementary Data Figure S4). The reference compound Donepezil exhibited a mean RMSD of 0.42 ± 0.18 Å, with a relatively wide range (0.00–0.79 Å) and the lowest stability score (0.846), compared to the top-ranked compounds.

Among the top-performing candidates, Z94570675 showed the lowest mean RMSD (0.21 ± 0.02 Å) and minimal deviation range (0.00–0.37 Å), corresponding to the highest stability score (0.976). This indicates a highly stable binding conformation with minimal structural perturbation throughout the simulation. Similarly, Z94570687 (0.28 ± 0.06 Å, stability score 0.947) and Z94570693 (0.35 ± 0.06 Å, stability score 0.945) also exhibited low RMSD values with limited fluctuations, suggesting consistent ligand positioning within the binding pocket. Compounds such as Z1824281875 and Z1498348710 maintained moderate RMSD values (0.38 ± 0.04 Å and 0.35 ± 0.05 Å, respectively) with high stability scores (>0.95), further supporting their stable interaction behavior.

**Figure 6:**
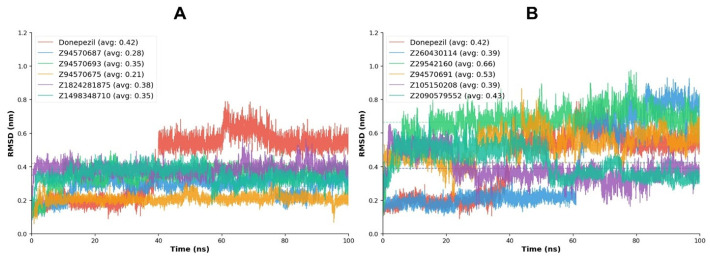
Time evolution of ligand-receptor RMSD (nm) over 100 ns molecular dynamics simulations. (**A**) Comparison of the reference inhibitor Donepezil with selected compounds (Z94570687, Z94570693, Z94570675, Z1824281875, and Z1498348710), showing relatively stable trajectories. (**B**) RMSD profiles of additional candidates (Z260430114, Z29542160, Z94570691, Z105150208, and Z2090579552) alongside Donepezil, indicating comparatively higher structural fluctuations for some complexes.

In the second group of compounds, the stability trends were slightly more variable. Z105150208 demonstrated the highest stability within this subset (mean RMSD 0.39 ± 0.08 Å, stability score 0.924), comparable to or slightly better than Donepezil. Compounds Z94570691 and Z2090579552 also showed acceptable stability, with moderate RMSD values and stability scores above 0.91, indicating relatively stable binding conformations. However, Z29542160 exhibited a higher mean RMSD (0.66 ± 0.10 Å) (Figure 6), suggesting increased structural deviations despite a reasonably good stability score (0.910). The least stable compound in this group was Z260430114, with a mean RMSD of 0.39 ± 0.25 Å and a wide fluctuation range (up to 0.93 Å), reflected in the lowest stability score (0.800), indicating significant conformational variability during the simulation.

Overall, the RMSD analysis demonstrates that several screened compounds exhibit superior or comparable stability to the reference compound Donepezil. In particular, Z94570675 stands out as the most stable candidate, followed by Z94570687 and Z94570693, all of which show consistently low RMSD values and minimal fluctuations. These findings suggest that the identified compounds not only possess strong binding affinity, as indicated by docking studies, but also maintain stable interactions within the binding site over time, reinforcing their potential as promising AChE inhibitors.

#### 2.9.2. Hydrogen Bond Analysis

**Figure 7:**
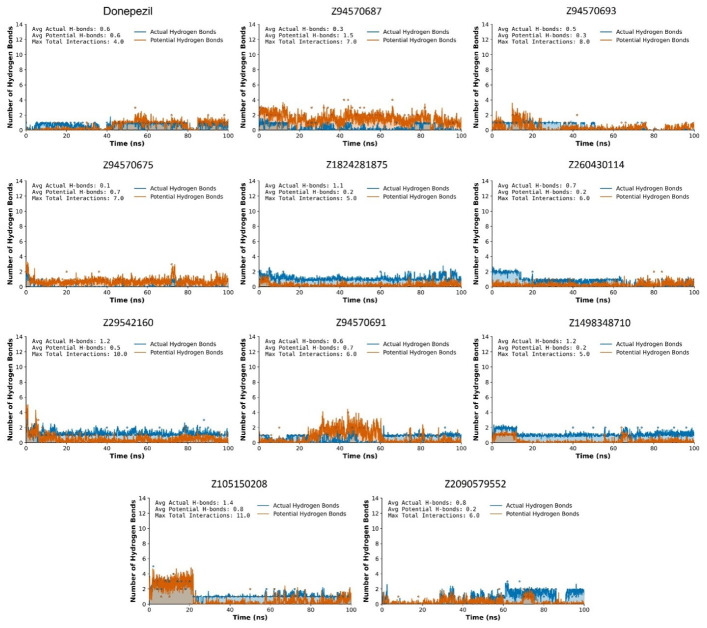
Time-dependent hydrogen bond (H-bond) analysis of protein–ligand complexes over 100 ns MD simulations. Consistent with prior BBB filtering, most compounds exhibit a low number of hydrogen bonds, indicating limited polar interactions while maintaining stable binding throughout the simulation.

The hydrogen bond interaction profiles of the selected simulated complexes over the 100 ns molecular dynamics simulations are presented in Figure 7. In this analysis, actual hydrogen bonds represent those satisfying strict geometric criteria for hydrogen bond formation, whereas potential hydrogen bonds correspond to interactions within a donor–acceptor distance cutoff of 0.35 nm, indicating close proximity that may facilitate transient or weak hydrogen bonding interactions. Interestingly, the compounds with excessive hydrogen bond donor and acceptor capacities were excluded during the earlier screening stages to ensure favorable BBB permeability. Despite this restriction, the selected compounds demonstrate sustained interaction with AChE through non-polar contacts complemented by occasional hydrogen bonding. The persistence of these interactions over the simulation time indicates stable ligand accommodation within the binding gorge.

The analysis reveals that the majority of the screened compounds maintain a low number of both stable (actual) and potential hydrogen bonds throughout the 100 ns simulation period. The consistently low values observed for both interaction types indicate that hydrogen bonding does not play a dominant role in ligand stabilization within the AChE binding pocket. Instead of relying on frequent hydrogen bond formation or even transient close-contact interactions (≤0.35 nm), the ligands exhibit minimal polar interactions overall. Despite this, the compounds remain well-positioned within the active site, suggesting that their binding is primarily governed by non-polar interactions such as hydrophobic contacts and π-based interactions with key aromatic residues.

Comparative analysis with the reference compound Donepezil reveals a similar trend, where relatively few stable hydrogen bonds are maintained. Taken together, the hydrogen bond analysis supports the earlier molecular interaction findings, confirming that the selected compounds maintain stable binding within the AChE active site while avoiding excessive polarity. This balance is critical for CNS drug candidates, as it enhances the likelihood of BBB penetration while preserving sufficient interaction strength for effective target inhibition.

#### 2.9.3. MD Interaction Energy

The interaction energy profiles of the selected AChE–ligand complexes were evaluated by decomposing the total interaction energy into short-range Coulombic (Coul-SR) and Lennard–Jones (LJ-SR) components. The total interaction energy represents the overall stability of ligand binding within the active site, with more negative values indicating stronger interactions. Overall, the results demonstrate that van der Waals (LJ-SR) interactions are the dominant contributors to ligand binding, while electrostatic (Coul-SR) interactions play a secondary role. This observation is consistent with the hydrophobic nature of the AChE binding gorge and aligns with the earlier molecular interaction and hydrogen bond analyses.

Compounds Z1824281875, Z1498348710, and Z94570687 exhibited the most favorable total interaction energies, with values of −62.72, −55.35, and −53.83 kcal/mol, respectively. Among these, Z1824281875 demonstrated the strongest binding, driven by both significant van der Waals (−41.95 kcal/mol) and electrostatic contributions (−20.77 kcal/mol). Similarly, Z1498348710 and Z94570687 showed enhanced LJ-SR contributions, indicating strong hydrophobic packing within the AChE active site (Table 4). These compounds clearly outperform the reference drug Donepezil, suggesting superior binding stability and optimal interaction complementarity.

**Figure 8:**
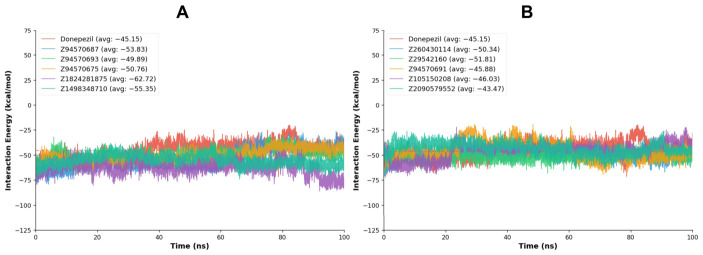
Decomposition of total MD interaction energy for the lead compounds in comparison with the reference compound across the 100 ns simulation. The top performing compounds are grouped in subfigure (**A**), while the moderate compounds are grouped in subfigure (**B**), in comparison with donepezil.

Compounds Z94570675, Z260430114, Z29542160, and Z94570693 fall into a moderate binding category (Figure 8), with total interaction energies ranging from approximately −49.88 to −51.81 kcal/mol. These compounds display a balanced contribution between Coul-SR and LJ-SR energies, although van der Waals interactions remain predominant. Their interaction energies are consistently more favorable than that of Donepezil (−45.15 kcal/mol), indicating improved binding affinity while maintaining similar interaction characteristics. Notably, Z29542160 shows a relatively stronger electrostatic contribution compared to others in this group.

Compounds Z94570691, Z105150208, and Z2090579552 exhibited comparatively weaker interaction energies, ranging from −43.47 to −46.03 kcal/mol. Despite this, their binding energies are still comparable to or slightly better than Donepezil. These compounds show reduced Coul-SR and LJ-SR contributions, suggesting less optimal packing or fewer stabilizing interactions within the binding pocket. However, their performance remains within an acceptable range, indicating potential as viable candidates. The reference inhibitor Donepezil demonstrated a total interaction energy of −45.15 kcal/mol, primarily driven by van der Waals interactions (−36.42 kcal/mol) with a smaller electrostatic contribution (−8.73 kcal/mol). Most of the screened compounds exhibited more favorable total interaction energies than Donepezil. This suggests that the identified compounds possess stronger binding affinity and improved interaction stability within the AChE active site.

### 2.10. Free-Energy Calculation

The binding free energies (ΔG_TOTAL_) of the AChE–ligand complexes were estimated using the MM/PBSA approach to quantify the thermodynamic stability of ligand binding. More negative ΔG values indicate stronger binding affinity and greater stability of the protein–ligand complex. The calculated energies ranged from −17.90 to −29.09 kcal/mol, with standard deviations between 4.03 and 5.66 kcal/mol, suggesting consistent and reliable sampling across the MD trajectories.

**Figure 9:**
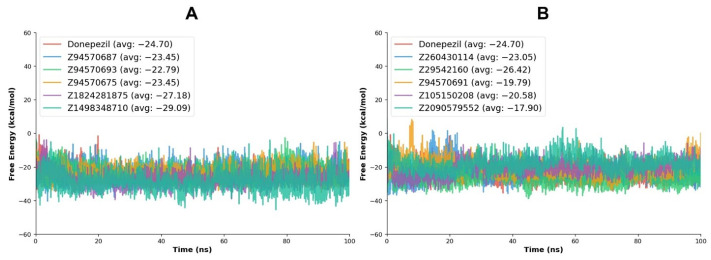
Binding free-energy calculations using the MM/PBSA method on trajectories extracted from the 100 ns molecular dynamics simulation. The top performing compounds are grouped in subfigure (**A**) in comparison with donepezil, while the moderate compounds are grouped in subfigure (**B**).

Compounds Z1498348710, Z1824281875, and Z29542160 exhibited the most favorable binding free energies, with ΔG values of −29.09, −27.18, and −26.42 kcal/mol, respectively (Table 5). Among these, Z1498348710 showed the strongest binding affinity, indicating highly stable complex formation within the AChE active site. These compounds significantly outperform the reference drug Donepezil (−24.70 kcal/mol), suggesting enhanced thermodynamic favorability and stronger ligand–protein interactions. Their relatively moderate standard deviations further support the stability of these interactions throughout the simulation. Furthermore, compounds Z94570687, Z94570675, Z260430114, and Z94570693 manifested the ΔG values ranging from −22.79 to −23.45 kcal/mol (Figure 9). Although slightly less favorable than Donepezil, these compounds still demonstrate strong binding affinities and stable interaction profiles. Their performance indicates a balanced interaction within the binding pocket, likely supported by dominant hydrophobic interactions, as observed in previous analyses. The relatively low standard deviations (≈4–5 kcal/mol) indicate consistent binding behavior over time.

Conversely, the compounds Z105150208, Z94570691, and Z2090579552 showed comparatively weaker binding free energies, with ΔG values of −20.58, −19.79, and −17.90 kcal/mol, respectively. These values are notably less favorable than Donepezil, suggesting reduced binding stability and weaker interaction strength within the AChE active site. Despite this, the compounds still maintain moderate interaction potential and may benefit from further optimization. Donepezil demonstrated a binding free energy of −24.70 kcal/mol. While it remains a strong inhibitor, several screened compounds, particularly top-ranked compounds, exhibited more favorable ΔG values, indicating improved binding affinity. This suggests that these compounds may serve as promising alternatives with potentially enhanced inhibitory activity.

The strong performance of top-ranked compounds highlights their potential as lead candidates for AChE inhibition. Furthermore, the consistency in standard deviation values across all systems indicates stable binding interactions throughout the simulation period. Importantly, the observed binding trends align with the dominance of hydrophobic interactions and limited hydrogen bonding within the AChE binding pocket. This not only supports strong ligand binding but also correlates with favorable BBB permeability profiles, making these compounds particularly suitable for CNS drug development.

### 2.11. In Vitro Activity Assay of AChE

**Figure 10:**
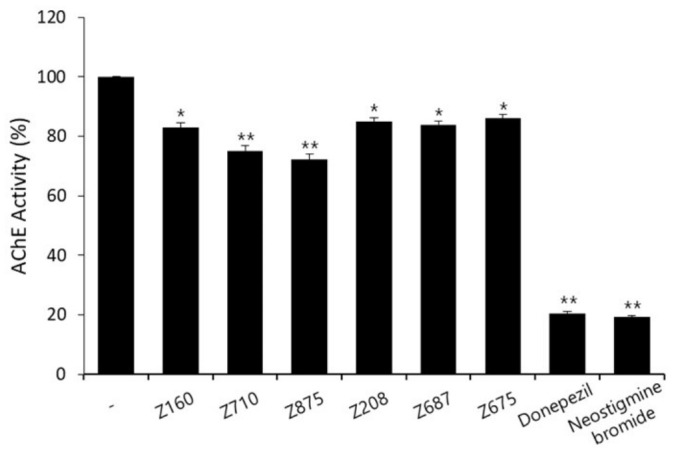
Inhibitory effects of test compounds on acetylcholinesterase (AChE) activity. Data are presented as mean ± SD (n = 3) and expressed as a percentage of the control group. Statistical significance was determined by Welch’s t-test compared to the control group (-) (* *p* < 0.05, ** *p* < 0.01).

To evaluate the inhibitory effects of the compounds on AChE activity, a fluorescence-based enzymatic assay was performed. As shown in Figure 10, all tested compounds exhibited varying degrees of AChE inhibition at a concentration of 50 μM. Among the tested compounds, Z1498348710 (Z710) and Z1824281875 (Z875) demonstrated relatively stronger inhibitory effects, reducing AChE activity to approximately 75% and 72% of the control levels, respectively (*p* < 0.01). In contrast, Z29542160 (Z160), Z105150208 (Z208), Z94570687 (Z687), and Z94570675 (Z675) showed detectable inhibition, with residual AChE activities ranging from 82% to 86% compared to the control group (*p* < 0.05). These results indicate that structural variations among the compounds significantly influence their inhibitory potency against AChE. The reference inhibitors, Donepezil and Neostigmine bromide, reduced AChE activity to approximately 20% of control levels (*p* < 0.01).

**Figure 11:**
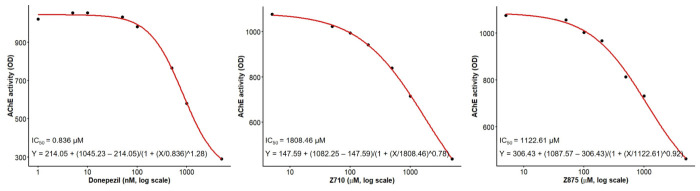
Dose–response curves and IC50 values of donepezil, Z710, and Z875 for acetylcholinesterase (AChE) inhibition.

To further characterize the two most active compounds, concentration-response studies were performed (Figure 11). The calculated IC_50_ values were 1808.46 μM for Z1498348710 (Z710) and 1122.61 μM for Z1824281875 (Z875), whereas the reference inhibitor Donepezil exhibited an IC_50_ of 835.89 nM. Although the compounds exhibited moderate inhibitory activity and IC_50_ against AChE compared to the reference inhibitors Donepezil and Neostigmine bromide in the in vitro assay. They represent novel chemical scaffolds identified through the integrated virtual screening workflow and may provide suitable starting points for subsequent lead optimization aimed at improving potency while maintaining favorable drug-like properties.

## 3. Discussion

The present study employed an integrated computational drug discovery workflow to identify novel acetylcholinesterase (AChE) inhibitors. Among the prioritized compounds, molecular docking demonstrated consistently favorable binding within the catalytic gorge of AChE. The identified compounds exhibited substantially lower CDocker energies (−33.15 to −43.64 kcal/mol) than the reference inhibitor Donepezil (−15.33 kcal/mol), suggesting energetically favorable binding conformations. Likewise, the CDocker interaction energies of the screened compounds (−43.37 to −58.59 kcal/mol) were comparable to or exceeded that of Donepezil (−56.06 kcal/mol), indicating that several compounds established extensive non-bonded interactions with residues lining the active site. These findings suggest that the newly identified scaffolds possess structural features capable of occupying the catalytic pocket and forming stable protein–ligand interactions comparable to established AChE inhibitors.

The stability of the docked complexes was further supported by molecular dynamics simulations. The majority of screened compounds maintained stable interactions throughout the simulation period, with total short-range MD interaction energies ranging from −43.47 to −62.72 kcal/mol, compared with −45.15 kcal/mol for Donepezil. In particular, Z1824281875 exhibited the strongest overall interaction energy (−62.72 kcal/mol), while Z1498348710 (−55.35 kcal/mol) and Z94570687 (−53.83 kcal/mol) also displayed stronger interactions than the reference inhibitor. These results indicate that the favorable docking poses remained energetically stable during dynamic simulation and were not artifacts of rigid receptor docking. Binding free-energy calculations using the MM-PBSA approach further supported these observations. Among all screened compounds, Z1498348710 exhibited the most favorable binding free energy (ΔG = −29.09 kcal/mol), followed by Z1824281875 (−27.18 ± 4.30 kcal/mol) and Z29542160 (−26.42 ± 4.31 kcal/mol), all of which demonstrated stronger predicted binding than Donepezil (−24.70 ± 4.09 kcal/mol). Most of the remaining compounds displayed binding free energies comparable to the reference inhibitor, suggesting that multiple candidates possess favorable thermodynamic interactions with the AChE binding pocket. Importantly, the agreement between molecular docking, MD interaction energies, and MM-PBSA calculations provides independent computational evidence supporting the stability and favorable binding characteristics of these newly identified scaffolds.

In addition to target binding, the pharmacokinetic characteristics of candidate compounds are critical for the development of central nervous system therapeutics. SwissADME analysis predicted that all selected compounds possess high gastrointestinal absorption, favorable oral bioavailability (0.55), and BBB permeability, indicating physicochemical properties compatible with oral administration and CNS exposure (Table 6). Notably, five of the six lead compounds were predicted not to be substrates of P-glycoprotein (P-gp), whereas Donepezil was classified as a P-gp substrate. Since P-gp-mediated efflux can reduce brain exposure of therapeutic agents, the absence of predicted P-gp recognition may represent a potentially favorable characteristic of these compounds. Nevertheless, these predictions should be interpreted cautiously because transporter interactions require experimental confirmation. The calculated synthetic accessibility scores (2.93–3.43) further suggest that the identified scaffolds remain chemically tractable and should be amenable to future medicinal chemistry optimization without excessive synthetic complexity.

Despite the consistently favorable computational predictions, the biological evaluation revealed only moderate inhibition of AChE at the tested concentration. Among the screened compounds, Z1498348710 and Z1824281875 exhibited the greatest inhibition, reducing enzyme activity to approximately 75% and 72% of the untreated control, respectively, whereas Donepezil reduced enzyme activity to approximately 20%. Furthermore, the experimentally determined IC_50_ values (1808.46 μM for Z1498348710 and 1122.61 μM for Z1824281875) were substantially higher than that of Donepezil (835.89 nM), confirming that the newly identified compounds possess moderate potency under the present assay conditions. The apparent discrepancy between computational predictions and experimental inhibition is not unexpected and highlights an important limitation of computational methods. Molecular docking evaluates the geometric compatibility and estimated interaction energy of a ligand within a protein binding pocket, whereas MM-PBSA estimates relative binding free energies based on sampled conformations during molecular dynamics simulations. Neither approach directly predicts enzyme inhibition kinetics or IC_50_ values. Biological potency is influenced by numerous additional factors, including ligand association and dissociation kinetics, catalytic transition-state stabilization, water-mediated interactions, entropic contributions, induced conformational changes beyond the simulation timescale, and assay-specific experimental conditions. Consequently, compounds predicted to bind favorably may not necessarily exhibit equivalent inhibitory potency in biochemical assays.

Previous studies have reported that Donepezil, although clinically effective, is associated with several limitations, including gastrointestinal adverse effects, dizziness, insomnia, bradycardia, and interpatient variability in therapeutic response [21,32,33]. These limitations continue to motivate the search for alternative AChE inhibitor scaffolds with improved pharmacological profiles. While the present study does not demonstrate superior efficacy over Donepezil in the biological assay, it successfully identifies novel, synthetically accessible scaffolds with favorable predicted drug-like properties and measurable biological activity. This study demonstrates that the identified compounds represent promising starting points for the development of next-generation AChE inhibitors rather than fully optimized therapeutic candidates. Systematic medicinal chemistry optimization guided by the structural information obtained from docking and molecular dynamics simulations may substantially improve inhibitory potency while preserving desirable physicochemical characteristics. In addition, future studies should evaluate the selectivity of these compounds against other cholinesterases and related esterases, particularly butyrylcholinesterase (BChE), to establish their target specificity, assess potential off-target effects, and further characterize their therapeutic potential.

## 4. Methodology

### 4.1. Dataset Collection and Preprocessing

A curated dataset of AChE inhibitors was retrieved from the ChEMBL database [34], comprising molecular structures represented as SMILES strings, ChEMBL identifiers, assay information, and corresponding bioactivity values (IC_50_). To ensure data reliability and consistency, only entries with experimentally validated IC_50_ measurements were retained for further analysis (Supplementary Data Figure S5). The dataset underwent a comprehensive preprocessing workflow, which involved the removal of incomplete, duplicate, or structurally invalid records, followed by filtering to exclusively include IC_50_-based activity data. Additionally, entries with non-positive or unreliable activity values (standard_value ≥ 0.1) were excluded to minimize noise and potential bias in subsequent modeling. To normalize the bioactivity data and make it suitable for regression-based machine learning models, IC_50_ values (expressed in nanomolar units) were transformed into their negative logarithmic form (pIC_50_), using the standard equation:

pIC_50_ = −log_10_(IC_50_ × 10^−9^) (1)

This transformation reduces data skewness and also facilitates better comparison across compounds with varying potency levels. Following normalization, the dataset was systematically ranked based on pIC_50_ values to prioritize compounds according to their inhibitory strength for downstream analyses, including visualization, validation, and interpretation of structure–activity relationships.

### 4.2. Molecular Descriptor and Feature Generation

Molecular features were computed using the RDKit library [35], enabling comprehensive characterization of the chemical properties of the compounds. Both physicochemical descriptors and molecular fingerprints were generated to capture structural and functional attributes relevant to acetylcholinesterase inhibition. The calculated descriptors included molecular weight (MW), lipophilicity (LogP), hydrogen bond acceptors (HBA), hydrogen bond donors (HBD), fraction of sp^3^-hybridized carbons (CSP3), number of rotatable bonds, number of rings, topological polar surface area (TPSA), and the number of aromatic rings. In addition to these descriptors, structural information was encoded using Morgan fingerprints with a radius of 2, resulting in a 2048-bit vector representation for each molecule. These descriptors and fingerprint features were subsequently combined into a single unified feature matrix, which served as the input for downstream machine learning analysis.

To better understand the chemical space represented by the dataset, exploratory data analysis was performed on the computed descriptors. Histogram plots were used to visualize the distribution of individual descriptors, providing insights into the range and diversity of molecular properties within the dataset. Furthermore, a correlation matrix was generated to assess relationships among the descriptors, facilitating the identification of potential redundancy and multicollinearity. This analysis helped ensure that the selected features provided meaningful and non-overlapping information for predictive modeling.

### 4.3. Machine Learning Model Development and Evaluation

The curated dataset comprised 6880 compounds represented by 2057 molecular features, including physicochemical descriptors and molecular fingerprint representations. The activity values exhibited a continuous distribution across the dataset without severe imbalance; therefore, no data resampling or balancing techniques were applied. Chemical diversity was assessed using Morgan fingerprints followed by principal component analysis (PCA), which demonstrated broad coverage of chemical space with multiple structural clusters, indicating that the dataset encompassed diverse molecular scaffolds suitable for machine learning model development.

The dataset was randomly partitioned into training (80%) and independent test (20%) subsets with the ratio of 80:20 using a fixed random seed (random_state = 42) to ensure reproducibility. The training set was used for model development, whereas the test set was reserved exclusively for validation. A Random Forest Regressor implemented in the scikit-learn library [33], was employed owing to its robustness, ability to model nonlinear structure–activity relationships, and resistance to overfitting in high-dimensional descriptor spaces. The model was developed using default parameters and trained on the combined feature set comprising both physicochemical descriptors and molecular fingerprint representations. Model performance was rigorously assessed on the test set using multiple statistical metrics to ensure comprehensive evaluation. This included Mean Squared Error (MSE = 0.84), Mean Absolute Error (MAE = 0.54), and the coefficient of determination (R^2^ = 0.65) to measure the linear relationship between predicted and experimental pIC_50_ values.

In addition to these quantitative metrics, scatter plots comparing predicted versus experimental pIC_50_ values were generated to provide a visual assessment of model accuracy and predictive consistency across the dataset. Moreover, Model robustness against chance correlations was evaluated through Y-randomization (response permutation testing). One hundred randomized models were generated by randomly permuting the experimental pIC_50_ values while maintaining the original descriptor matrix. The randomized models produced a mean R^2^ of −0.005, confirming that the predictive performance of the original model was not attributable to random correlations (Supplementary Data Figure S6). Furthermore, the applicability domain (AD) of the final model was investigated using the Williams plot, where leverage values and standardized residuals were calculated for all compounds. The warning leverage (h*) was determined as 0.897, and the majority of compounds were located within the accepted applicability domain, with standardized residuals between ±3, indicating that the model provides reliable predictions for most compounds within the represented chemical space.

### 4.4. Virtual Screening of External Library

A large external screening library derived from the ChEMBL database was processed for activity prediction using the trained model. To efficiently handle the substantial size of the dataset and optimize memory usage, molecular descriptors and fingerprint features were computed in a batch-wise manner. Predictions of pIC_50_ values were subsequently generated using a chunk-based processing approach, ensuring computational scalability and stability. The screened compounds were then ranked according to their predicted pIC_50_ values to prioritize potential AChE inhibitors. Compounds exhibiting predicted pIC_50_ values greater than 7 were classified as high-confidence hits. To ensure uniqueness and avoid redundancy, duplicate entries were removed based on SMILES representations, and the top-ranked compounds were selected for further visualization and analysis.

### 4.5. Protein Structure Retrieval and Binding Pocket Characterization

The three-dimensional structure of AChE was obtained from the Protein Data Bank (PDB) [36] using the crystal structure with PDB ID: 7E3H, which has a resolution of 2.45 Å [37]. The retrieved structure was prepared for analysis by removing non-essential molecules such as water and heteroatoms, while retaining the co-crystallized ligand to facilitate accurate identification of the active site. Structural validation and secondary structure composition were assessed using VADAR v1.8 [38], which provided information on helices, β-sheets, coils, and turns, as well as backbone dihedral angle distributions. The stereochemical quality of the protein structure was further evaluated through Ramachandran plot analysis to confirm the reliability of residue conformations.

For binding pocket characterization, the active site of AChE was defined based on the coordinates of the co-crystallized ligand present in the crystal structure. The binding cavity was examined to identify key amino acid residues involved in ligand recognition and stabilization. Residue interactions within the binding pocket were analyzed to determine the nature of ligand–protein interactions, including hydrogen bonding, hydrophobic contacts, and van der Waals interactions. Particular attention was given to conserved aromatic residues lining the active-site gorge, as well as residues directly contributing to catalytic activity.

### 4.6. Molecular Docking

Molecular docking was performed to evaluate the binding modes and predicted binding affinities of the top 72 high-ranked compounds obtained from the prior screening workflow, along with the reference inhibitor Donepezil. All docking calculations were carried out using the CDocker protocol implemented in BIOVIA Discovery Studio v22 [39], which employs a CHARMm force field-based molecular dynamics simulated annealing algorithm to generate energetically favorable ligand conformations within the receptor binding site. Prior to docking, both ligand and receptor structures were systematically prepared to ensure the accuracy and reliability of the docking predictions.

Ligand were processed using the Ligand Preparation module, including energy minimization, generation of relevant tautomeric and stereoisomeric forms, assignment of protonation states at physiological pH, and correction of structural or valence inconsistencies. The AChE crystal structure was prepared using the Receptor Preparation protocol by adding missing hydrogen atoms, optimizing protonation states at pH 7.5, correcting incomplete side chains and loop regions where necessary, and performing energy minimization with the CHARMm force field using an energy convergence criterion of 0.9 [40,41,42,43,44]. The docking site was defined using the position of the co-crystallized ligand present in the experimental AChE structure. A binding sphere centered at X = −43.7952, Y = 38.1018, and Z = −30.2824 with a radius of 9.7766 Å was generated to encompass the entire catalytic binding pocket. The same binding sphere and docking parameters were subsequently used for both docking validation and screening of all candidate compounds, thereby ensuring methodological consistency.

To validate the docking protocol, the co-crystallized ligand was first removed from the receptor structure and subsequently redocked into the original binding site using the identical docking parameters described above. Ten independent binding poses were generated and superimposed with the experimental crystallographic binding conformation. All redocked poses reproduced the native binding orientation within the active site and occupied the same binding pocket as the co-crystallized ligand, confirming that the selected docking parameters accurately reproduced the experimentally observed binding mode (Supplementary Data Figure S7). Following successful validation, the optimized docking protocol was applied to the 72 prioritized compounds and the reference inhibitor Donepezil. Multiple binding poses were generated for each ligand and ranked according to the CDocker interaction energy and CDocker energy scores.

### 4.7. BBB Permeability Assessment

The BBB permeability of candidate compounds was evaluated using a consensus physicochemical scoring model based on molecular properties that have consistently been identified as key determinants of passive BBB penetration. Unlike data-driven machine learning BBB prediction models, this approach was developed as a transparent knowledge-based prioritization strategy using experimentally validated physicochemical principles reported in the literature [45,46,47,48,49,50,51,52]. The selected descriptors included topological polar surface area (TPSA), hydrogen bond donors (HBD), lipophilicity (LogP), hydrogen bond acceptors (HBA), and molecular weight (MW), which are among the most widely recognized molecular determinants governing BBB permeability.

The weighting assigned to each descriptor was based on its relative importance reported in previous studies. TPSA was assigned the largest contribution (35%) because numerous investigations have demonstrated that molecular polarity is one of the strongest determinants of passive diffusion across the BBB, with compounds exhibiting TPSA values below approximately 60–90 Å^2^ generally displaying improved brain penetration [45,46]. HBD (30%) were considered the second most influential descriptor, as increasing hydrogen-bonding capacity substantially decreases membrane permeability and BBB transport [47,48]. LogP contributed 20% because an optimal balance between aqueous solubility and membrane partitioning is essential for CNS exposure, whereas excessive lipophilicity may increase nonspecific tissue binding and metabolic instability [49,50]. HBA (10%) [51], and MW (5%) [49] were included as secondary contributors because they have been shown to influence BBB permeability, although generally to a lesser extent than molecular polarity and hydrogen-bonding characteristics.

In this framework, compounds with lower TPSA values (≤60 Å^2^) were considered highly favorable, while increasing TPSA resulted in progressively reduced contributions to the final score. Similarly, compounds with fewer hydrogen bond donors (HBD = 0) were prioritized, as excessive hydrogen bonding reduces membrane permeability. LogP values within the range of 2–3.5 were considered optimal for passive diffusion across the BBB, with moderate contributions assigned to slightly broader ranges. HBA and MW were also incorporated as secondary descriptors, favoring compounds with HBA ≤ 6 and MW within 350–450 Da, consistent with the established CNS drug-like space. In addition to the physicochemical scoring, compounds were further filtered based on their ionization state. Molecules containing permanent charges were excluded, as such species are generally unable to cross the BBB via passive diffusion [53]. This additional filtering criterion further increased the probability of selecting compounds with physicochemical characteristics compatible with CNS exposure.

### 4.8. Molecular Dynamics Simulation

Protein–ligand complexes exhibiting the most favorable docking energy and BBB permeability scores were selected for further investigation through 100 ns molecular dynamics (MD) simulations to assess their structural stability and dynamic interaction profiles within the AChE active site. All simulations were conducted using the CHARMM36 force field [54,55], with system preparation and input file generation performed via the CHARMM-GUI interface [56] to ensure compatibility with the GROMACS package [57].

Each complex was embedded in a cubic periodic simulation box and solvated using the TIP3P explicit water model [57]. System neutrality was achieved by adding appropriate counter-ions. Periodic boundary conditions were applied in all three spatial dimensions to mimic physiological conditions. Short-range non-bonded interactions, including van der Waals and electrostatic forces, were calculated using the Verlet cutoff scheme with a cutoff distance of 10 Å, while long-range electrostatic interactions were treated using the Particle Mesh Ewald (PME) method to ensure accurate representation of Coulombic interactions. Bond constraints involving hydrogen atoms were maintained using the LINCS algorithm, enabling stable integration of the equations of motion.

Prior to the production phase, each system was subjected to energy minimization using the steepest descent algorithm to eliminate steric clashes and unfavorable contacts. This was followed by equilibration in two sequential steps: constant volume and temperature (NVT) equilibration, followed by constant pressure and temperature (NPT) equilibration to stabilize thermodynamic parameters such as temperature, pressure, and density. The equilibrated systems were then used for production MD simulations carried out for 100 ns with a time step of 2 fs using GROMACS version 2022.

### 4.9. Free-Energy Calculation

The binding free energies of the AChE–ligand complexes were estimated using the Molecular Mechanics/Poisson–Boltzmann Surface Area (MM/PBSA) method as implemented in gmx_MMPBSA (version 1.6.3), based on trajectories generated from molecular dynamics (MD) simulations [58,59]. To ensure robust and statistically meaningful results, the complete 100 ns production trajectories were utilized for the calculations without any frame selection or truncation.

For each system, free-energy calculations were performed for three distinct components: the solvated protein–ligand complex, the isolated receptor, and the free ligand in solution. The binding free energy (ΔG_TOTAL_) was computed using the following thermodynamic relationship:

ΔG_TOTAL_ = G_complex_ − (G_protein_ + G_ligand_) (2)

where G_complex_, G_protein_, and G_ligand_ represent the total free energies of the complex, the receptor, and the ligand, respectively, in the solvent environment. This approach combines molecular mechanics energy terms with solvation energies to provide an overall estimate of the binding affinity. All MM/PBSA calculations were performed using the same force field parameters and solvent models employed during the MD simulations to maintain consistency across the computational workflow. The final binding free-energy values were obtained by averaging the computed ΔG values over the entire simulation trajectory for each protein–ligand system.

### 4.10. Acetylcholinesterase (AChE) Activity Assay

Acetylcholinesterase (AChE) activity was determined by SensoLyte^®^ 520 acetylcholinesterase activity assay kit (AS-72242; AnaSpec, Fremont, CA, USA) with minor modifications to the manufacturer’s protocol. Test compounds (Z160, Z710, Z875, Z208, Z687, and Z675) were purchased from Enamine (Kyiv, Ukraine). Reference inhibitor, Donepezil was obtained from MedChemExpress (HY-14566; Monmouth Junction, NJ, USA). Briefly, AChE enzyme was pre-incubated with each compound at a final concentration of 50 μM at 37 °C for 1 h. Following pre-incubation, the fluorogenic substrate provided in the kit was added to initiate the reaction. The reaction mixtures were incubated at 37 °C for an additional 1 h in a 96-well microplate. Fluorescence intensity was measured using a microplate reader (Spectra ax M5, Molecular Devices, San Jose, CA, USA) with excitation and emission wavelengths set at 490 nm and 520 nm, respectively. AChE activity was expressed as a percentage relative to the untreated control group.

#### 4.10.1. Statistical Analysis

All experiments were performed independently at least three times, and the data are presented as the mean ± standard deviation (SD). Statistical analyses were performed using R (version 4.5.1; R Foundation for Statistical Computing, Vienna, Austria). Comparisons between each treatment group and the corresponding untreated control group were conducted using Welch’s two-sample *t*-test, which does not assume equal variances between groups. Differences were considered statistically significant at *p* < 0.05, with significance levels indicated as *p* < 0.05 (*) and *p* < 0.01 (**).

#### 4.10.2. Determination of IC50 Values for AChE Inhibition

The half-maximal inhibitory concentration (IC_50_) values of donepezil, Z710, and Z875 against AChE were determined from concentration-response curves. Donepezil was tested at concentrations of 0, 0.0001, 0.0005, 0.01, 0.05, 0.1, 0.5, 1, and 5 μM, whereas Z710 and Z875 were evaluated at concentrations of 0, 5, 50, 100, 200, 500, 1000, and 5000 μM. Because logarithmic transformation cannot be applied to zero, the 0 μM control was excluded from the nonlinear regression analysis but was retained as the reference control for AChE activity. Dose–response curves were fitted using a four-parameter logistic (4PL) nonlinear regression model implemented in the drc package in R (version 4.5.1; R Foundation for Statistical Computing, Vienna, Austria) within the RStudio (v2025.05.01, Build 513) environment, and IC_50_ values were estimated from the fitted curves.

## 5. Conclusions

In the present study, an integrated computational workflow combining machine learning, BBB permeability screening, molecular docking, molecular dynamics simulations, and MM-PBSA calculations was employed to prioritize potential AChE inhibitors from the large Enamine chemical library, followed by experimental validation of the top-ranked candidates. The multi-step screening strategy efficiently identified several compounds with favorable predicted binding characteristics, stable protein–ligand interactions, and predicted drug-like physicochemical properties suitable for further investigation. Biological evaluation confirmed that the selected compounds possess measurable AChE inhibitory activity, with Z1498348710 and Z1824281875 exhibiting the greatest inhibition among the screened candidates. Although these compounds were considerably less potent than the reference inhibitor Donepezil, the experimental results demonstrate that the computational workflow was successful in enriching compounds with genuine biological activity. Importantly, the identified compounds exhibited favorable predicted drug-like properties and predicted physicochemical characteristics desirable for CNS drug discovery. The structural diversity of these scaffolds provides valuable opportunities for medicinal chemistry optimization aimed at improving inhibitory potency while preserving their desirable drug-like characteristics.

## Figures and Tables

**Table 1:** Feature importance analysis of the machine learning model showing the contribution of molecular fingerprints and physicochemical descriptors. The top 10 most influential fingerprint bits are also listed, highlighting key structural features driving model predictions.

Feature Importance, Including Fingerprints				Top 10 Fingerprint Bits		
Sr	Feature	Importance	Percentage	Sr	Finger Prints	Importance
1	Fingerprints (Total)	0.823073	82.274200	1	FP_712	0.0934
2	LogP	0.038779	3.876306	2	FP_800	0.0632
3	MW	0.034082	3.406822	3	FP_1214	0.0575
4	CSP3	0.030480	3.046797	4	FP_1064	0.0506
5	NumRotBond	0.029928	2.991613	5	FP_1048	0.0292
6	TPSA	0.017560	1.755295	6	FP_1506	0.0152
7	NumRings	0.010565	1.056084	7	FP_1564	0.0121
8	HBA	0.005882	0.587943	8	FP_314	0.0108
9	HBD	0.005561	0.555889	9	FP_522	0.0081
10	NumAromaticRings	0.004090	0.408876	10	FP_1970	0.0081
11	Fingerprints (Mean)	0.000402	0.040173			

**Table 2:** CDocker docking results of the top-ranked compounds against AChE, showing CDocker energy and interaction energy values.

Sr No.	Catalog ID	CDocker Energy (kcal/mol)	CDocker Interaction Energy (kcal/mol)
1	Z94570687	−43.6447	−52.6065
2	Z94570693	−43.3405	−55.8720
3	Z94570675	−43.0556	−52.8524
4	Z1824281875	−42.9042	−56.8551
5	Z260430114	−40.5476	−53.9189
6	Z29542160	−40.0182	−55.6579
7	Z94570691	−39.7155	−53.1723
8	Z1498348710	−37.4084	−58.5919
9	Z105150208	−35.8450	−52.1556
10	Z2090579552	−35.5308	−44.9759
11	Z212833264	−34.9263	−55.1516
12	Z238483938	−33.9650	−55.9929
13	Z86312003	−33.7623	−43.3713
14	Z1348443492	−33.5735	−47.4713
15	Z2047906229	−33.1509	−52.4869
16	Donepezil	−15.3345	−56.0576

**Table 3:** Blood–brain barrier (BBB) permeability assessment of the top-ranked compounds, including predicted pIC_50_ values, BBB scores, and charge properties. All selected compounds satisfied BBB criteria and demonstrated favorable CNS penetration potential.

Sr No.	Catalog_ID	pIC_50_	BBB_Score	Has_Permanent_Charge	BBB_Pass_Fail
1	Z94570687	7.3874	1	FALSE	PASS
2	Z94570693	7.1379	1	FALSE	PASS
3	Z94570675	7.3685	1	FALSE	PASS
4	Z1824281875	7.2837	0.95	FALSE	PASS
5	Z260430114	7.2608	0.95	FALSE	PASS
6	Z29542160	7.1595	1	FALSE	PASS
7	Z94570691	7.1076	1	FALSE	PASS
8	Z1498348710	7.0131	0.95	FALSE	PASS
9	Z105150208	7.2472	0.98	FALSE	PASS
10	Z2090579552	7.3304	0.9	FALSE	PASS
11	Donepezil	7.0232	0.85	FALSE	PASS

**Table 4:** MD interaction energy analysis of the protein–ligand complexes, showing short-range Coulombic (Coul-SR), Lennard–Jones (LJ-SR), and total interaction energies.

Sr No.	Compound	Interaction Energy (kcal/mol)		
		Coul-SR	LJ-SR	Total Energy
1	Z1824281875	−20.7687	−41.9503	−62.7189
2	Z94570693	−10.5702	−39.3179	−49.8880
3	Z94570675	−11.7791	−38.9775	−50.7566
4	Z260430114	−9.9240	−40.4113	−50.3353
5	Z94570687	−10.8890	−42.9398	−53.8288
6	Z29542160	−15.6119	−36.1986	−51.8105
7	Z94570691	−10.5540	−35.3253	−45.8793
8	Z1498348710	−13.2316	−42.1138	−55.3454
9	Z105150208	−13.9842	−32.0476	−46.0317
10	Z2090579552	−7.6507	−35.8176	−43.4684
11	Donepezil	−8.7265	−36.4221	−45.1486

**Table 5:** MM/PBSA binding free-energy calculations of the protein–ligand complexes, along with standard deviations, indicate the stability and binding affinity of the selected compounds.

Sr	Compounds	ΔG_TOTAL_	Standard Deviation
1	Z94570687	−23.45	4.62
2	Z94570693	−22.79	4.4
3	Z1824281875	−27.18	4.3
4	Z94570675	−23.45	4.03
5	Z260430114	−23.05	5.09
6	Z29542160	−26.42	4.31
7	Z94570691	−19.79	4.98
8	Z1498348710	−29.09	5.66
9	Z105150208	−20.58	4.48
10	Z2090579552	−17.90	4.67
11	Donepezil	−24.70	4.09

**Table 6:** The predicted physicochemical properties of the top-ranked compounds using SwissADME online webserver.

Compounds	Oral Bioavailability	P-gp Substrate	GI Absorption	BBB Permeant	Synthetic Accessibility
Z94570687	0.55	No	High	Yes	3.04
Z1824281875	0.55	No	High	Yes	3.43
Z94570675	0.55	Yes	High	Yes	3.28
Z29542160	0.55	No	High	Yes	3.26
Z1498348710	0.55	No	High	Yes	2.93
Z105150208	0.55	No	High	Yes	3.05
Donepezil	0.55	Yes	High	Yes	3.36

## Data Availability

The original contributions presented in this study are included in the article. Further inquiries can be directed to the corresponding author.

## Supplementary Materials

The following supporting information can be downloaded at: https://www.mdpi.com/article/10.3390/ph19071120/s1, Figure S1: The correlation matrix of molecular descriptors of the reference data set; Figure S2: The predicted pIC50 values of the target enamine library; Figure S3: The 2D structural representation of the screened top 25 Enamine compounds with the pIC50 values. Figure S4: Backbone RMSD profiles of AChE–ligand complexes during 100 ns molecular dynamics simulations. (A) Donepezil and compounds Z94570687, Z94570693, Z94570675, Z1824281875, and Z1498348710; (B) Donepezil and compounds Z260430114, Z29542160, Z94570691, Z105150208, and Z2090579552, with the apo AChE backbone shown in gray; Figure S5: The conversion and standardization of the reference data into pIC50 values; Figure S6: (A) PCA of the screening library reveals broad chemical space coverage across multiple structural clusters. (B) Y-randomization confirms the model’s predictive R^2^, validating against chance correlation. (C) Distribution of pIC_50_ values. (D) Williams’ plot showing the applicability domain of the model; Figure S7: Redocking validation of the co-crystallized ligand. (A) Superimposition of the docked ligand and the co-crystallized ligand within the receptor binding site. (B) Enlarged view showing the ten generated docked conformations overlaid with the co-crystallized ligand.
