# Supplementary material for: Machine Learning and Molecular Modeling Strategy for the Identification of CNS-Active Acetylcholinesterase Inhibitors
Source: Pharmaceuticals (Basel). 2026 Jul 20;19(7):1120. doi: 10.3390/ph19071120 (PMC13415056; doi:10.3390/ph19071120)
Supplement: Supplementary file 1 [file pharmaceuticals-19-01120-s001.zip › pharmaceuticals-4403477-supplementary.pdf]

Article

Machine Learning and Molecular Modeling Strategy for the Identification of CNS-Active Acetylcholinesterase Inhibitors

Muhammad Yasir [1], Jinyoung Park [1], Eun-Taek Han [2], Won Sun Park [3], Jin-Hee Han [2], Jongseon Choe [4] and Wanjoo Chun [1,*]

[1]Department of Pharmacology, Kangwon National University School of Medicine, Chuncheon, 24341, Republic of Korea;

[2]Department of Medical Environmental Biology and Tropical Medicine, Kangwon National University School of Medicine, Chuncheon, 24341, Republic of Korea;

[3]Department of Physiology, Kangwon National University School of Medicine, Chuncheon, 24341, Republic of Korea;

[4]Department of Microbiology and Immunology, Kangwon National University School of Medicine, Chuncheon, 24341, Republic of Korea

[*]Correspondence: author: Dr. Wanjoo Chun, Department of Pharmacology Kangwon National University School of Medicine, Kangwon National University, Email: wchun@kangwon.ac.kr, Phone: +82-33-250-8853.

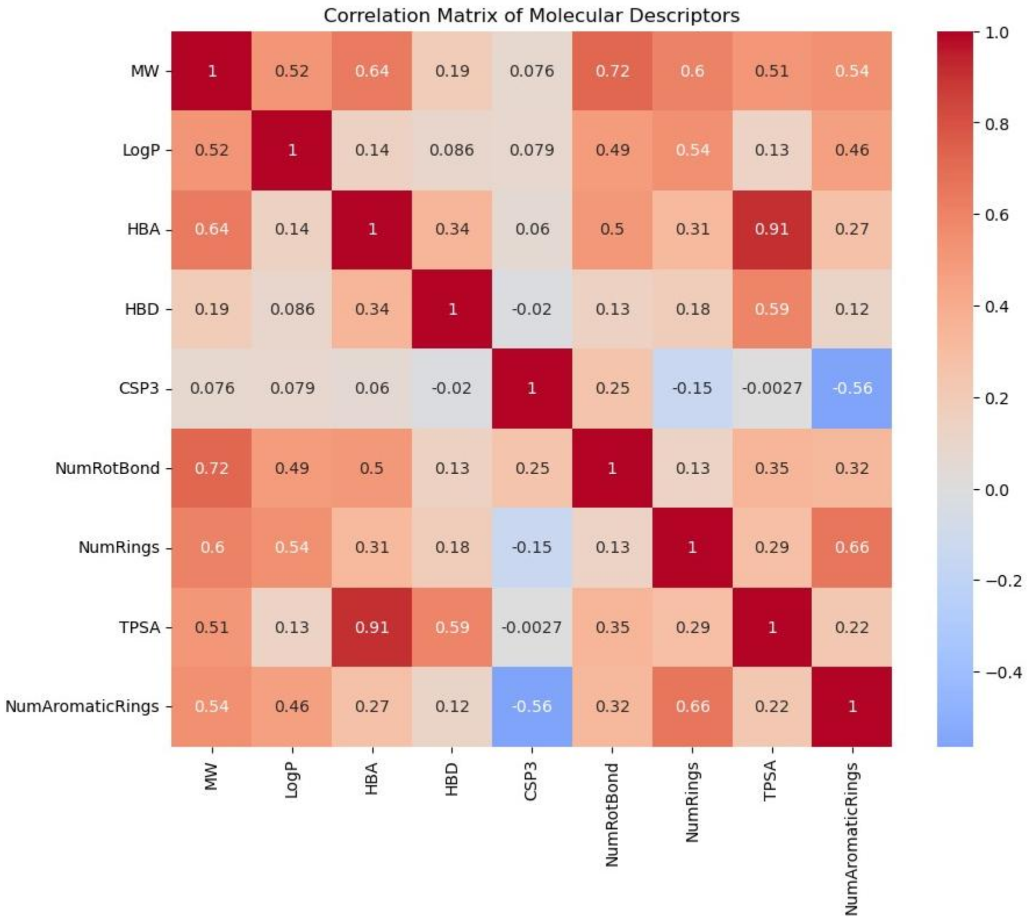

Figure S1. The correlation matrix of molecular descriptors of the reference data set.

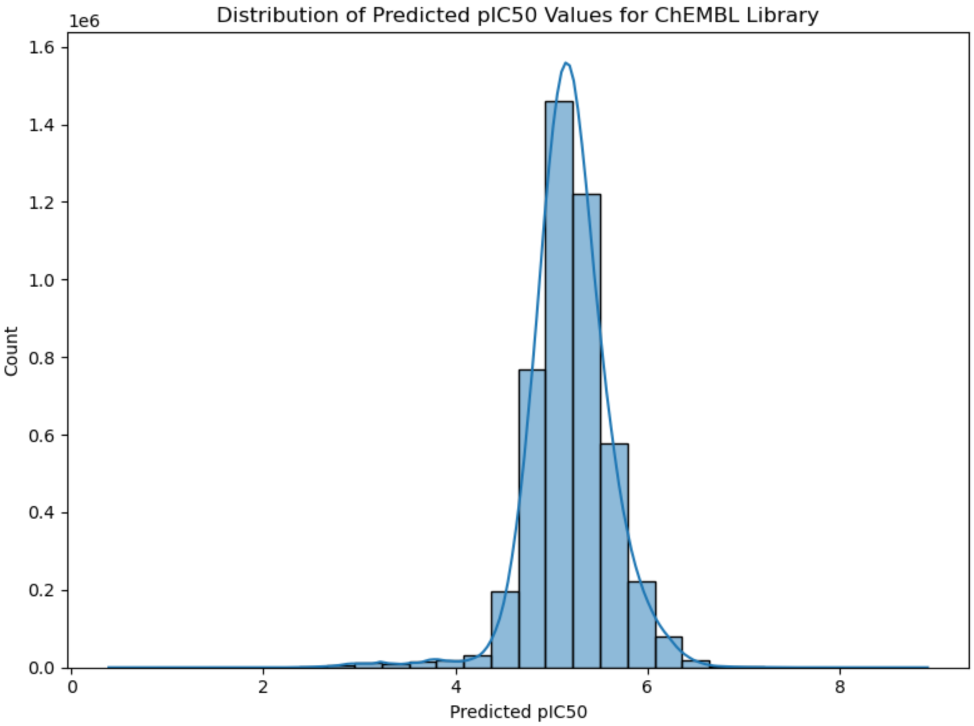

Figure S2. The predicted pIC50 values of the target enamine library.

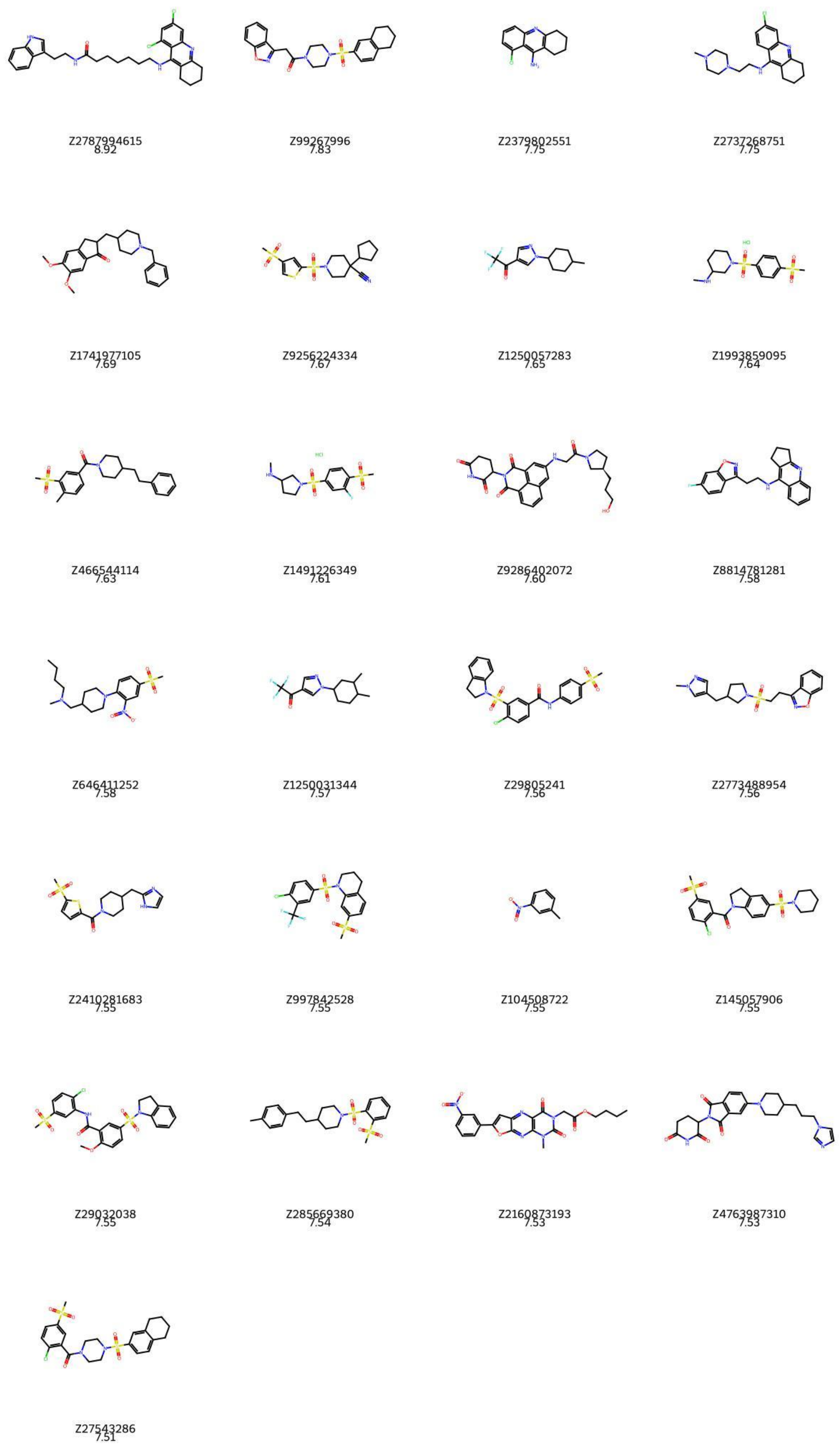

Figure S3. The 2D structural representation of the screened top 25 Enamine compounds with the pIC50 values.

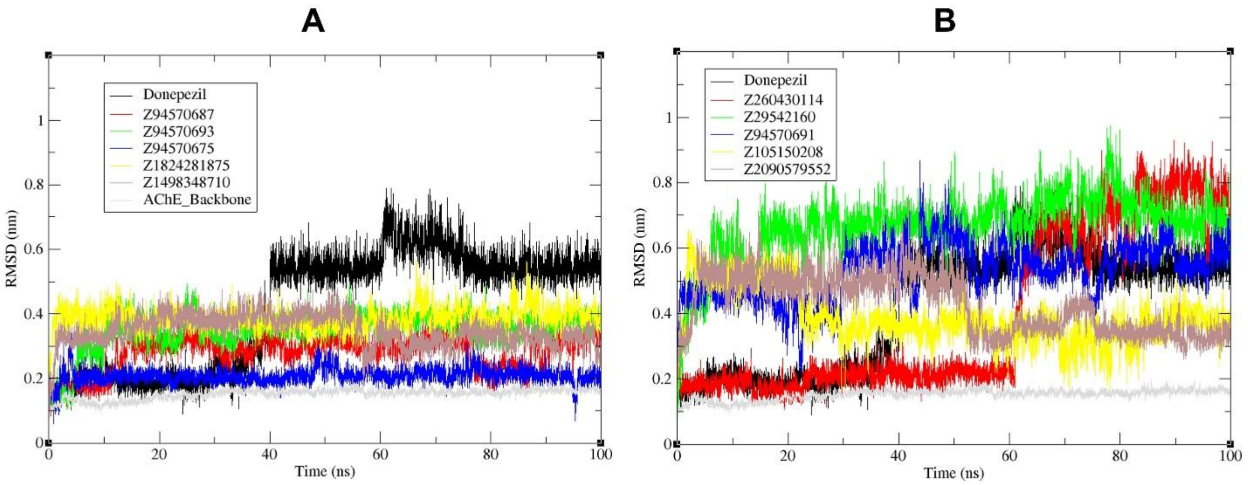

Figure S4. Backbone RMSD profiles of AChE–ligand complexes during 100 ns molecular dynamics simulations. (A) Donepezil and compounds Z94570687, Z94570693, Z94570675, Z1824281875, and Z1498348710; (B) Donepezil and compounds Z260430114, Z29542160, Z94570691, Z105150208, and Z2090579552, with the apo AChE backbone shown in gray.

	canonical_smiles	molecule_chembl_id	standard_type	standard_value	pIC50
7496	COc1ccc2oc(C(=O)NCCCCCCCCCNc3c4c(nc5cc(Cl)ccc...	CHEMBL2019035	IC50	1.000000e-01	10.000000
2289	O=C(CCCCCCNc1c2c(nc3cc(Cl)ccc13)CCCC2)NCCc1c[n...	CHEMBL381499	IC50	1.000000e-01	10.000000
2619	O=C(Cc1c[nH]c2ccccc12)NCCCCCCNc1c2c(nc3cc(Cl)...	CHEMBL223443	IC50	1.200000e-01	9.920819
14903	COc1cc2c(cc1O)CCC(C)(CCNCCCCCCCCNc1c3c(nc4cc(C...	CHEMBL4751100	IC50	1.200000e-01	9.920819
2623	O=C(CCCc1c[nH]c2ccccc12)NCCCCCNc1c2c(nc3cc(Cl)...	CHEMBL225021	IC50	1.300000e-01	9.886057
...	...	...	...	...	...
217	COc1ccccc1CNCCCCCC(=O)NCCCCCCCCNC(=O)CCCCCNCc1...	CHEMBL134488	IC50	5.370320e+14	-5.730000
214	COc1ccccc1CNCCCCCC(=O)N(C)CCCCCCCCN(C)C(=O)CCC...	CHEMBL132377	IC50	3.235940e+15	-6.510000
216	Nc1c2c(nc3ccccc13)CCCC2	CHEMBL95	IC50	4.570880e+15	-6.660000
207	COc1ccccc1CN(C)CCCCCC(=O)N(C)CCCCCCCCN(C)C(=O)...	CHEMBL11805	IC50	5.888440e+15	-6.770000
210	CNC(=O)Oc1ccc2c(c1)[C@]1(C)CCN(C)[C@@H]1N2C	CHEMBL94	IC50	7.079460e+16	-7.850000

6880 rows × 5 columns

Figure S5. The conversion and standardization of the reference data into pIC50 values.

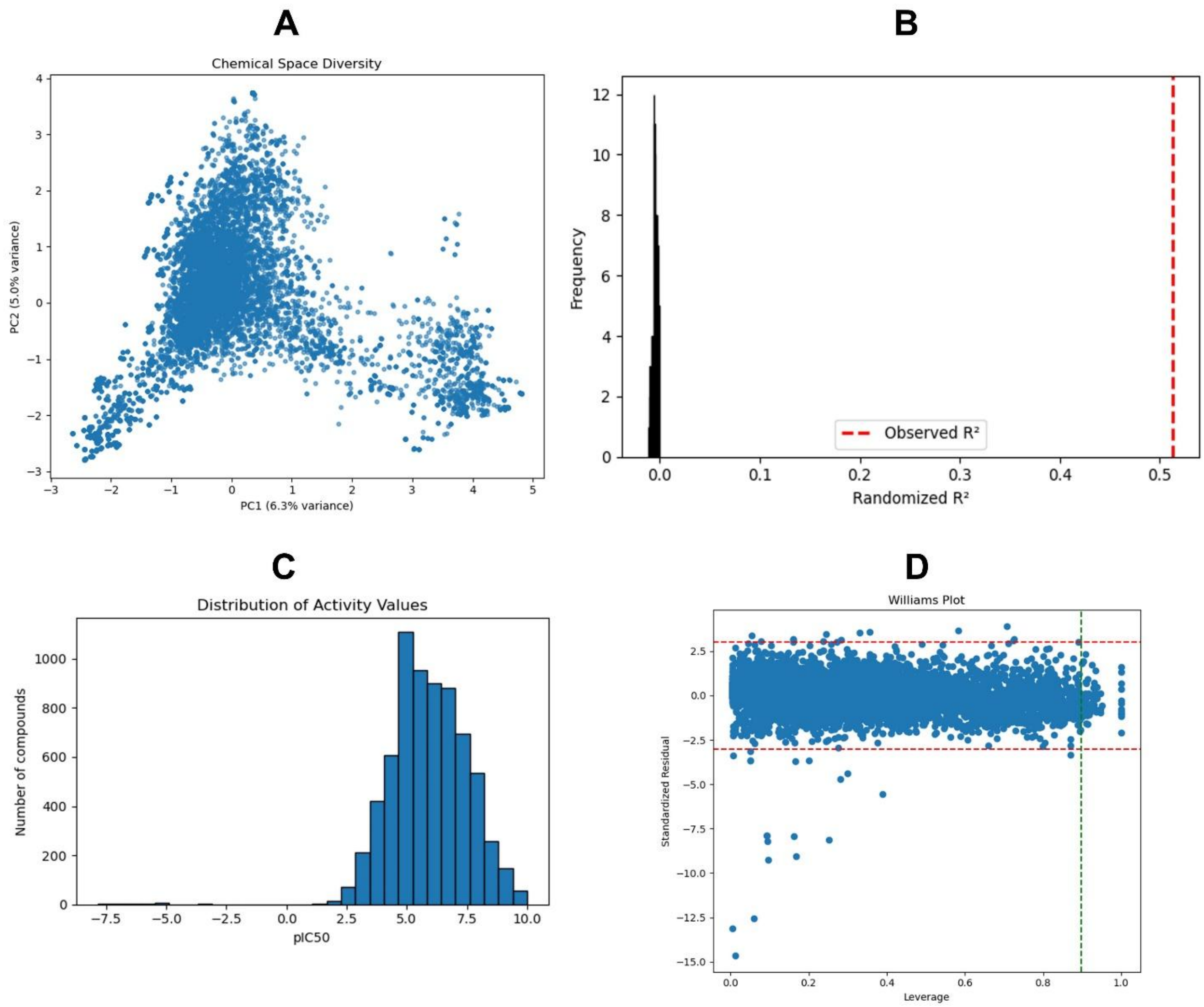

Figure S6. (A) PCA of the screening library reveals broad chemical space coverage across multiple structural clusters. (B) Y-randomization confirms the model's predictive R^2, validating against chance correlation. (C) Distribution of pIC_{50} values. (D) Williams' plot showing the applicability domain of the model.

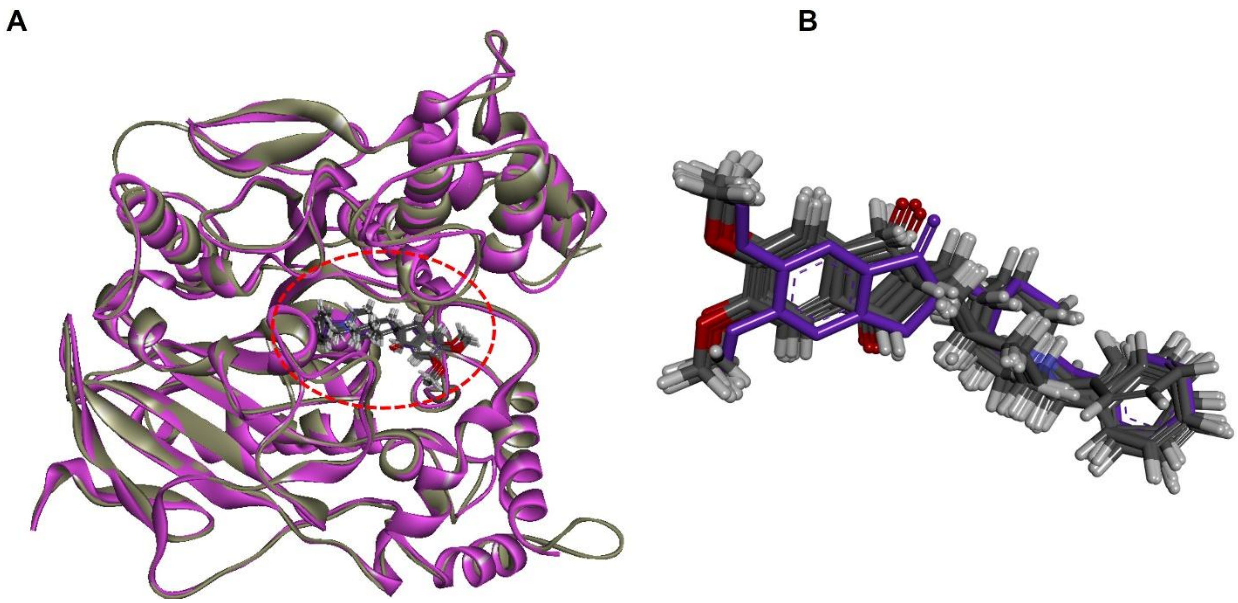

Figure S7. Redocking validation of the co-crystallized ligand. (A) Superimposition of the docked ligand and the co-crystallized ligand within the receptor binding site. (B) Enlarged view showing the ten generated docked conformations overlaid with the co-crystallized ligand.
